# A Novel Nonpedicular Screw-Based Fixation in Lumbar Spondylolisthesis

**DOI:** 10.1155/2017/5619350

**Published:** 2017-01-10

**Authors:** Ming-Hong Chen, Jen-Yuh Chen

**Affiliations:** ^1^Division of Neurosurgery, Department of Surgery, Cathay General Hospital, Taipei, Taiwan; ^2^School of Medicine, Catholic Fu-Jen University, New Taipei City, Taiwan; ^3^Department of Orthopedic Surgery, Taipei Medical University Hospital, Taipei, Taiwan; ^4^Department of Orthopedics, School of Medicine, College of Medicine, Taipei Medical University, Taipei, Taiwan; ^5^Taipei Postal Hospital, Taipei, Taiwan

## Abstract

*Objective*. The authors present the clinical results obtained in patients who underwent interspinous fusion device (IFD) implantation following posterior lumbar interbody fusion (PLIF). The purpose of this study is investigating the feasibility of IFD with PLIF in the treatment of lumbar spondylolisthesis.* Methods*. Between September 2013 and November 2014, 39 patients underwent PLIF and subsequent IFD (Romeo®2 PAD, Spineart, Geneva, Switzerland) implantation. Medical records of these patients were retrospectively reviewed to collect relevant data such as blood loss, operative time, and length of hospital stay. Radiographs and clinical outcome were evaluated 6 weeks and 12 months after surgery.* Results*. All 39 patients were followed up for more than one year. There were no major complications such as dura tear, nerve injuries, cerebrospinal fluid leakage, or deep infection. Both interbody and interspinous fusion could be observed on radiographs one year after surgery. However, there were 5 patients having early retropulsion of interbody fusion devices.* Conclusion*. The interspinous fusion device appears to achieve posterior fixation and facilitate lumbar fusion in selected patients. However, further study is mandatory for proposing a novel anatomic and radiological scoring system to identify patients suitable for this treatment modality and prevent postoperative complications.

## 1. Introduction

Lumbar arthrodesis with decompression of the neural structures is an effective surgical management for degenerative spondylolisthesis with stenosis. The current methods for lumbar arthrodesis include posterolateral fusion, posterior interbody fusion (PLIF), and transforaminal lumbar interbody fusion (TLIF) with pedicle screw instrumentation. However, these treatment modalities involving the pedicle screw-based fixation have several drawbacks. The most common complications associated with pedicle screw fixation included unrecognized screw misplacement, fracturing of the pedicle, and iatrogenic cerebrospinal fluid leak. Consequently, mechanical failure, transient neurapraxia, or permanent nerve root injury could happen [1, 2]. In addition, postoperative back pain could result from wide muscle dissection and long operative times for pedicular screw fixation.

Recently, interspinous spacers have been developed to satisfy the requirements of minimally invasive procedures, decrease the morbidity associated with pedicle screw instrumentation, and prevent the overload on adjacent vertebral segments. As the growing applications of interspinous devices, the surgical indications have been extended, ranging in degenerative stenosis, discogenic low back pain, herniated intervertebral disc diseases, and low-grade instability [3]. Interspinous process devices can be categorized by design as static, dynamic, or fusion devices. The intention of all these implants is designed to maintain certain distraction between the spinous processes. Nevertheless, the development of interspinous fusion devices (IFD) is intended to be an alternative to pedicle screw fixation system and to aid in the stabilization of the spine with interbody fusion. Theoretically, using IFD instead of pedicle screw fixation inherits the advantages of other interspinous process devices such as delivery through a single incision, reduced disruption of paraspinal musculature, and reduced risk of nerve injuries [4]. In addition, adjacent segmental degeneration (ASD) resulted from pedicle screw [5–7] and rod fixation may be prevented by less rigid fixation provided by IFD. When IFD are used in combination with interbody fusion devices, they potentially offer a biomechanically circumferential fusion comparable or improved outcomes compared to traditional pedicle screw and rod instrumentation. Therefore, in this study, we retrospectively reviewed our experience with 39 patients who underwent the interspinous fusion devices (IFD) combined with posterior lumbar interbody fusion (PLIF) for grade I lumbar spondylolisthesis.

## 2. Materials and Methods

Between September 2013 and November 2014, 39 patients with grade I lumbar spondylolisthesis underwent posterior IFD (Romeo 2 PAD, Spineart, Geneva, Switzerland) and posterior lumbar interbody fusion (Juliet®OL, Spineart, Geneva, Switzerland). The demographic data of the patients is listed on Table 1.

The study group included 39 patients, 6 males and 33 females, who ranged in age from 52 to 79 years (mean 66.0 years) at the time of surgery. All patients included in this study presented with radicular pain and/or intermittent claudication and required laminoforaminotomy for neural decompression. Levels of lumbar spondylolisthesis in this study were as follows: L3-4 in 9 patients, L4-5 in 27 patients, L3-4-5 in 2 patients, and L2-3 and L4-5 in 1 patient. L4-5 spondylolisthesis was most common. Patients with advanced spondylolisthesis (≥grade II spondylolisthesis) were excluded from this study. Other exclusion criteria included (1) osteoporosis; (2) disabling leg from compression fracture, metabolic neuropathy, or vascular claudication; (3) previous surgery at the intended treatment level.

**Figure 1:**
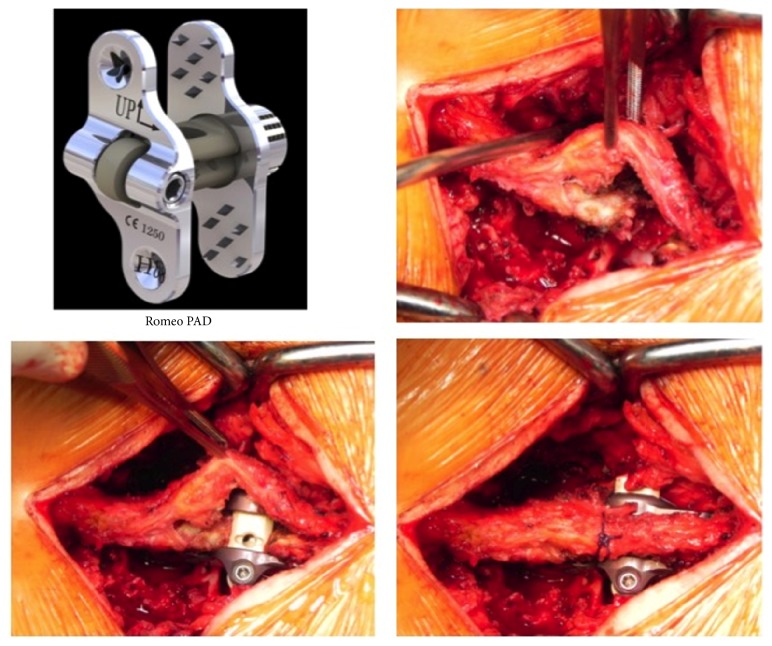
Implantation of interspinous fusion devices (Romeo 2 PAD, Spineart, Geneva, Switzerland).

All patients underwent PLIF with a poly-ether-ether-ketone (PEEK) cage (Juliet OL, Spineart, Geneva, Switzerland) and interspinous fusion with an IFD composed of two titanium plates with 30 degrees' polyaxiality and a PEEK central core (Romeo 2 PAD, Spineart, Geneva, Switzerland). A 4-5 cm midline skin incision was made at the intended fusion level overlying the spinous processes. Exposure of the rostral and caudal spinous processes was done by routine subperiosteal dissection with preservation of the supraspinous ligament for later anatomical closure. Dissection was carried out down to the lumbar lamina. Decompression with insertion of an interbody cage was done over the symptomatic side using standard PLIF procedures. Both autograft harvested from laminoforaminotomy and artificial bone graft were packed in the interbody cage and adjacent to the cage in the disc space to provide larger contact area for bone fusion. The interspinous ligament was removed to facilitate implantation of IFD. A small portion of the edge of rostral and caudal spinous processes was removed to expose cancellous bone. The central PEEK core of IFD was filled with autologous bone fragments harvested from the decompression procedures before implantation of IFD. Trial spacers of different sizes were inserted in the interspinous space to select IFD of optimal size. Once the two plates of selected IFD were inserted with one plate on each side of the spinous processes, the easy one-step locking mechanism allowed us to compress, fix, and lock the implant between the rostral and caudal portions of spinous processes. The supraspinous ligament was secured to the spinous processes for anatomical restoration (Figure 1). Finally, the wound was closed using standard multilayered methods without drainage. All patients were asked to wear orthosis for three months after surgery and avoid bending and lifting heavy objects.

Medical records of 39 patients were reviewed to collect data such as estimated blood loss (EBL), operative time, and length of hospital stay. The clinical outcome including back pain and sciatica was measured using the visual analogue scale (VAS). All outcome measures were assessed on the day after surgery for the immediate postoperative outcome and 6 weeks and 12 months after surgery. Postoperative radiographs including plain anteroposterior and flexion-extension views were evaluated at 6 weeks and 12 months after surgery.

## 3. Results

There were a total of 6 men and 33 women in the study. The mean operative time for interspinous fusion and posterior interbody fusion ranged from 65 to 105 minutes, which was considerably less than that in the open pedicle screw fixation. The mean estimated blood loss ranged from 120 ml to 150 ml in all 39 patients. The average length of hospital stay was 5.5 days in average. There were no intraoperative complications such as dura tear and nerve injury in all 39 patients.

**Figure 2:**
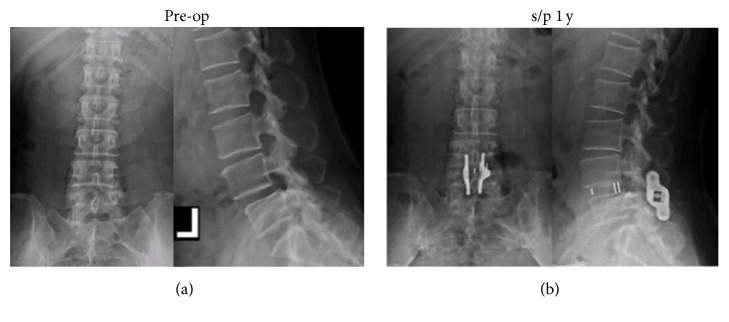
Imaging studies obtained in a 59-year-old female who presented with grade I spondylolisthesis (a). The patient underwent interspinous fusion device implantation following PLIF. One year after surgery, anteroposterior and lateral radiographs confirmed the position of the IFD and cage and showed both interbody and interspinous bone fusion (b).

The follow-up duration was over one year in all 39 patients. Immediately postoperatively, all patient experienced improvement in both sciatica and back pain. Except for 5 patients developing early migration of interbody devices, the remaining patients had favorable outcome in sciatica and back pain during one-year follow-up. Mean leg pain (VAS) decreased from 7.2 to 3.1 and 2.2, 6 weeks and 12 months after surgery, respectively. Interbody fusion was observed in 34 patients one year after surgery. Interestingly, interspinous fusion could also be observed in anteroposterior radiographs (Figure 2). There was no adjacent segment degeneration in our patients.

**Figure 3:**
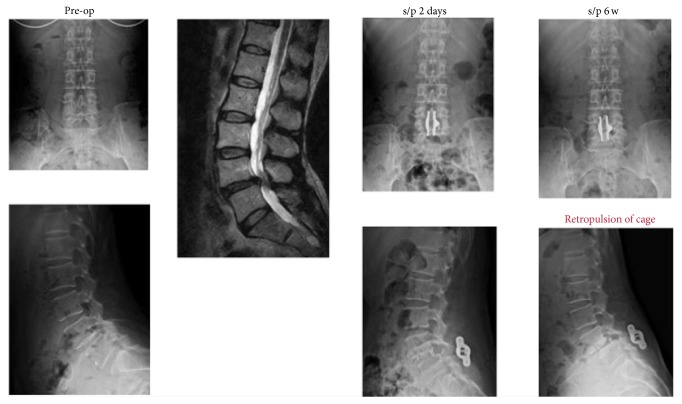
An illustrative 59-year-old female case demonstrated grade I spondylolisthesis on preoperative image studies. Radiographs 2 days after surgery showed IFD and interbody cage in appropriate positions. However, early migration of interbody cage was noted on the lateral radiograph 6 weeks after surgery.

There were five patients (3 female, 2 male, age ranged from 52 to 79 years) suffering from early retropulsion of interbody devices.  Figure 3 showed an illustrative 57-year-old woman of early retropulsion of IFD. However, fracture of the spinous processes or migration of interspinous devices did not happen in any patient. There was no major surgery-related complication such as deep infection, nerve root injury, and CSF leakage in our patients.

## 4. Discussion

The aim of interspinous process devices is to neutralize excessive movement in flexion and extension associated with distraction of the spinal segments to opening of the foramens [8]. Development of various IPD was attempted to reduce the risks associated with the pedicle screw fixation. 4% cerebrospinal fluid leakage, 2% transient neurapraxia, 2% permanent nerve root injury, 4-5% deep infection, and 3–12% hardware failure have been reported for the pedicle screw fixation technique [1, 2]. In addition, superior segment facet violation has recently shown to cause the adjacent level destabilization in pedicle screw fixation [9]. In this study, IFD implants were placed on only the spinous processes and presented no risk of dural or neural injury and cerebrospinal fluid leakage. Furthermore, the operative time for IFD implantation was shorter than that for the pedicle screw fixation. Additionally, smaller incision, minimal bone exposure, and less muscle retraction/dissection decreased the blood loss for IFD implantation. Because many patients receiving lumbar fusion surgery are elderly, lowering the blood loss during surgery indicates reduced surgical risks and better postoperative recovery.

Although previous study showed that range of motion (ROM) at the instrumented level was significantly decreased in both the IFD and pedicle screw fixation compared with the preoperative state [10], 12.8% of our patients (5 in 39 patients) developed posterior migration of the interbody cage during the early postoperative period (6 weeks). This result necessitates the reexamination of biomechanical characteristics of the interspinous devices. Hartmann et al. evaluated biomechanical effect of different interspinous devices on lumbar spinal range of motion. The results showed that interspinous devices led to a significant reduction in ROM during flexion-extension but to a significant increase in ROM during lateral bending and rotation [11]. A biomechanical study using human cadaver spines conducted by Techy et al. also demonstrated no statistically significant difference in the ROM in flexion-extension among the stand-alone interspinous devices and unilateral and bilateral pedicle fixation constructs. However, in both lateral bending and axial rotation, the unilateral and bilateral pedicle screw fixation constructs were significantly more rigid than the IPD alone and the interbody device combination [12]. Contrarily, Wang and associates reported interspinous devices (Spire SPP; Medtronic, Minneapolis, MN, USA) could limit axial rotation and lateral bending ROM as well as unilateral pedicle screw and rod fixation [13]. Another in vitro biomechanical study suggested that both bilateral pedicle screw fixation and IFD provided equivalent flexion-extension and axial rotation stability in a posterior lumbar interbody fusion model with posterior expandable cages [14]. Without doubt, a stand-alone interbody device allows facet sliding which can potentially prevent spinal arthrodesis. It is preferred to include posterior augmentation to simulate 360-degree stability. However, the exact amount of stiffness and ROM required to promote arthrodesis is ambiguous. Besides, the stability provided by instrumentation must cross an unknown threshold, varying from patient to patient, to ensure fusion. In spite of the increasing use of interspinous implants, the 2011 clinical guidelines from the NASS (North American Spine Society) suggested that there is insufficient evidence to support the net benefit for long-term outcomes for the placement of an interspinous process device [15, 16]. In addition, complications associated with interspinous process device implantation for lumbar spine degenerative disease included fracture of the spinous process, implant dislocation, and dura tear with cerebrospinal fluid (CSF) leakage. An European multicenter study revealed the complication rate was 7.8%. The ultimate failure rate requiring additional surgery was 9.6% [3]. Compared with IPD, clinical experience for IFD to date is limited; a previous report suggested the results of IFD investigations were promising in posterior lumbar interbody fusion models compared to select cases of bilateral pedicle screw fixation [10]. Wang et al. compared a small group (21 patients) with an interspinous device (Spire SPP; Medtronic, Minneapolis, MN, USA) used to supplement interbody fusion to 11 patients with bilateral pedicle screws. They demonstrated less blood loss and shorter operative time, without an increase in the rate of pseudarthrosis or hardware failure in the interspinous device group [17]. However, there was a high extrusion/expulsion rate of interbody devices in our patients. Interestingly, in our series, there was no incidence of cage subsidence, which has been frequently reported in stand-alone cage implantation [18]. Therefore, interspinous fusion devices as a posterior augmentation for spinal stability may contribute to the avoidance of cage subsidence in our study. Nevertheless, a deliberate preoperative anatomic and radiologic scoring system should be developed for patient selection to avoid migration of interbody cage. Because there was no consensus that had been reached on defining or classifying spondylolisthesis with respect to its stability, we were not able to determine the causes of posterior migration of the interbody cages from our cases. However, a degenerative lumbar spondylolisthesis instability classification (DSIC) proposed by Simmonds et al. [19] including the analysis of disc angle, presence of joint effusion, and signs of restabilization may provide preoperative evaluations for better patient selection for this IFD treatment modality in the future.

## 5. Conclusion

Bilateral pedicle screw stabilization with interbody body fusion is still considered the gold standard in lumbar arthrodesis. Posterior interspinous fusion device for one-level fusion is associated with minimal operative risk of dura and nerve injuries, shortens operative time, and decreases intraoperative blood loss. The interspinous fusion device also appears to achieve posterior fixation and facilitate lumbar fusion in selected patients. However, it is not a panacea for all patients with lumbar spondylolisthesis. Future study for preoperative evaluation and analysis of anatomic/radiological pitfalls and tips is mandatory for proposing a novel scoring system to identify patients suitable for this treatment modality and prevent postoperative complications.

## Figures and Tables

**Table 1:** Demographic data in patients undergoing PLIF with IFD.

Patients	IFD
Number	39
Sex (male : female)	6 : 33
Age	52–79 (mean 66.0)
Spondylolisthesis level	Number of cases
L3-4	9
L4-5	27
L3-4-5	2
L2-3 and L4-5	1
